# Feasibility of the optimal cerebral perfusion pressure value identification without a delay that is too long

**DOI:** 10.1038/s41598-022-22566-6

**Published:** 2022-10-22

**Authors:** Mantas Deimantavicius, Edvinas Chaleckas, Katherine Boere, Vilma Putnynaite, Tomas Tamosuitis, Arimantas Tamasauskas, Mindaugas Kavaliauskas, Saulius Rocka, Aidanas Preiksaitis, Saulius Vosylius, Solventa Krakauskaite, Kristina Berskiene, Vytautas Petkus, Arminas Ragauskas

**Affiliations:** 1 Health Telematics Science Institute, Kaunas University of Technology, K. Barsausko Str. 59, 51423 Kaunas, Lithuania; 2 Department of Intensive Care, Academy of Medicine, Lithuanian University of Health Sciences, Eiveniu Str. 2, 50161 Kaunas, Lithuania; 3 Department of Applied Mathematics, Kaunas University of Technology, Studentų Str. 50-319, 51367 Kaunas, Lithuania; 4 Clinic of Neurology and Neurosurgery, Faculty of Medicine, Vilnius University, Santariskiu Str. 2, 08661 Vilnius, Lithuania; 5 Clinic of Anaesthesiology and Intensive Care, Faculty of Medicine, Vilnius University, Siltnamiu Str. 29, 04130 Vilnius, Lithuania; 6 Department of Sports Medicine, Lithuanian University of Health Sciences, Tilzes Str. 18, 47181 Kaunas, Lithuania

**Keywords:** Biophysics, Neuroscience, Health care, Medical research, Mathematics and computing

## Abstract

Optimal cerebral perfusion pressure (CPPopt)-targeted treatment of traumatic brain injury (TBI) patients requires 2–8 h multi-modal monitoring data accumulation to identify CPPopt value for individual patient. Minimizing the time required for monitoring data accumulation is needed to improve the efficacy of CPPopt-targeted therapy. A retrospective analysis of multimodal physiological monitoring data from 87 severe TBI patients was performed by separately representing cerebrovascular autoregulation (CA) indices in relation to CPP, arterial blood pressure (ABP), and intracranial pressure (ICP) to improve the existing CPPopt identification algorithms. Machine learning (ML)-based algorithms were developed for automatic identification of informative data segments that were used for reliable CPPopt, ABPopt, ICPopt and the lower/upper limits of CA (LLCA/ULCA) identification. The reference datasets of the informative data segments and, artifact-distorted segments, and the datasets of different clinical situations were used for training the ML-based algorithms, allowing us to choose the appropriate individualized CPP-, ABP- or ICP-guided management for 79% of the full monitoring time for the studied population. The developed ML-based algorithms allow us to recognize informative physiological ABP/ICP variations within 24 min intervals with an accuracy up to 79% (compared to the initial accuracy of 74%) and use these segments for timely optimal value identification or CA limits determination in CPP, ABP or ICP data. Prospective clinical studies are needed to prove the efficiency of the developed algorithms.

## Introduction

The maintenance of patient-specific cerebral perfusion pressure (CPP) to ensure a stable and adequate cerebral blood flow is important for improving the outcome of traumatic brain injury (TBI) patients^1–3^. The Brain Trauma Foundation guidelines recommend a target CPP range of 60–70 mmHg to prevent secondary injuries caused by ischemia or increased edema^4^. However, this universal target range for CPP may not be the optimal for the diverse population affected by TBI. Many factors can influence the individual optimal CPP range, including age, elevated intracranial pressure (ICP), brain injury severity, glucose levels, pharmaceutical treatment, and brain trauma severity^5^.

Optimal cerebral perfusion pressure (CPPopt)-targeted management was proposed as a possible solution for personalized patient treatment for severe TBI and subarachnoid hemorrhage (SAH) cases^6–9^. This type management is based on controlling the CPP and maintaining it at patient-specific CPPopt value, thus avoiding cerebrovascular autoregulation (CA) impairments and secondary brain insults.

The benefit of CPPopt-targeted management to improve TBI/SAH patient outcomes was analyzed in pilot and retrospective studies^1,5–12^ as well as in phase II clinical trials^2,13,14^. It was demonstrated that CPP deviation from the identified CPPopt value is associated with worse patient outcomes and the maintaining the CPP within +/− 5–10 mmHg of CPPopt value is a strategy that yields for more favorable patients outcomes^8,11,12,14,15^. Larger deviations from CPPopt value outside the patient-specific limits are associated with CA impairment, leading to ischemia, hyperemia and, consequently, to unfavorable outcomes^15^.

The ICM + software (Cambridge Enterprise Ltd., UK) is used in TBI/SAH clinical trials^1,2,5–14^ for the identification of the CPPopt value. Typically requires 4 or more hours of continuous monitoring of high-resolution ICP(t) and arterial blood pressure (ABP(t)) data, continuous CA assessment by calculating the pressure reactivity index (PRx(t)), and determination of the CPPopt value at the best intact CA condition are required. PRx(t) values are identified as a moving Pearson correlation coefficient between ICP(t) and ABP(t) slow waves within a 5 min or larger moving average window^14^. The negative PRx(t) values represent intact CA, while the positive PRx(t) values represent impaired CA. Ideally, plotting PRx against CPP will generate a “U-shaped” curve with a minimum point indicating the CPPopt value as a target for a personalized treatment of the TBI patient^6,7^.

However, many factors still limit the practical application of CPPopt-targeted treatment.

The identification of PRx(t) requires the presence of slow ABP(t) and ICP(t) waves to reliably assess CA status. Slow intracranial waves have an intermittent nature with random variations in amplitude and oscillation frequency^16^. PRx values are close to zero if the amplitude of slow waves is too low. Diagnostic information is lost in such cases^17^. The “U-shaped” curve when plotting PRx against CPP is obtained only when the CPP varies in a wide enough range (e.g., from the lower to the upper autoregulation limits) and the PRx values are identified without unacceptable uncertainty in the presence of slow ABP(t) and ICP(t) waves of appropriate amplitude. Otherwise, a “U-shaped” curve cannot be obtained, or only a part of this curve can be identified. The quality of the “U-shaped” curve is also affected if the monitoring data used for CPPopt identification includes artifacts. All these factors can significantly affect the quality of the informative data required to identify the CPPopt value. Nevertheless, even with a 4-h ABP(t)/ICP(t) data accumulation window, the CPPopt value can be identified in approximately 50–60% of the monitoring time. For some patients, CPPopt value identification is impossible^18^.

Recently, a new adaptive multiwindow weighted approach for CPPopt value identification was proposed allowing the possibility of identifying CPPopt values within up to 89% of the full monitoring time^19^. This approach is based on the adaptive extension of the data accumulation time window from 2- to 8-h until the CPPopt value is identified^18,19^. However, such a long data accumulation and processing time are associated with delayed CPPopt-guided TBI management. Clinical evidence suggests that cerebrovascular impairment lasting longer than 40–60 min is associated with a risk of brain damage, thus impacting TBI patients outcomes^5,20^. We need to identify CPPopt values in less than 2- to 8-h of monitoring data accumulation to implement a patient-specific CPPopt-targeted therapy that is clinically effective.

**Figure 1:**
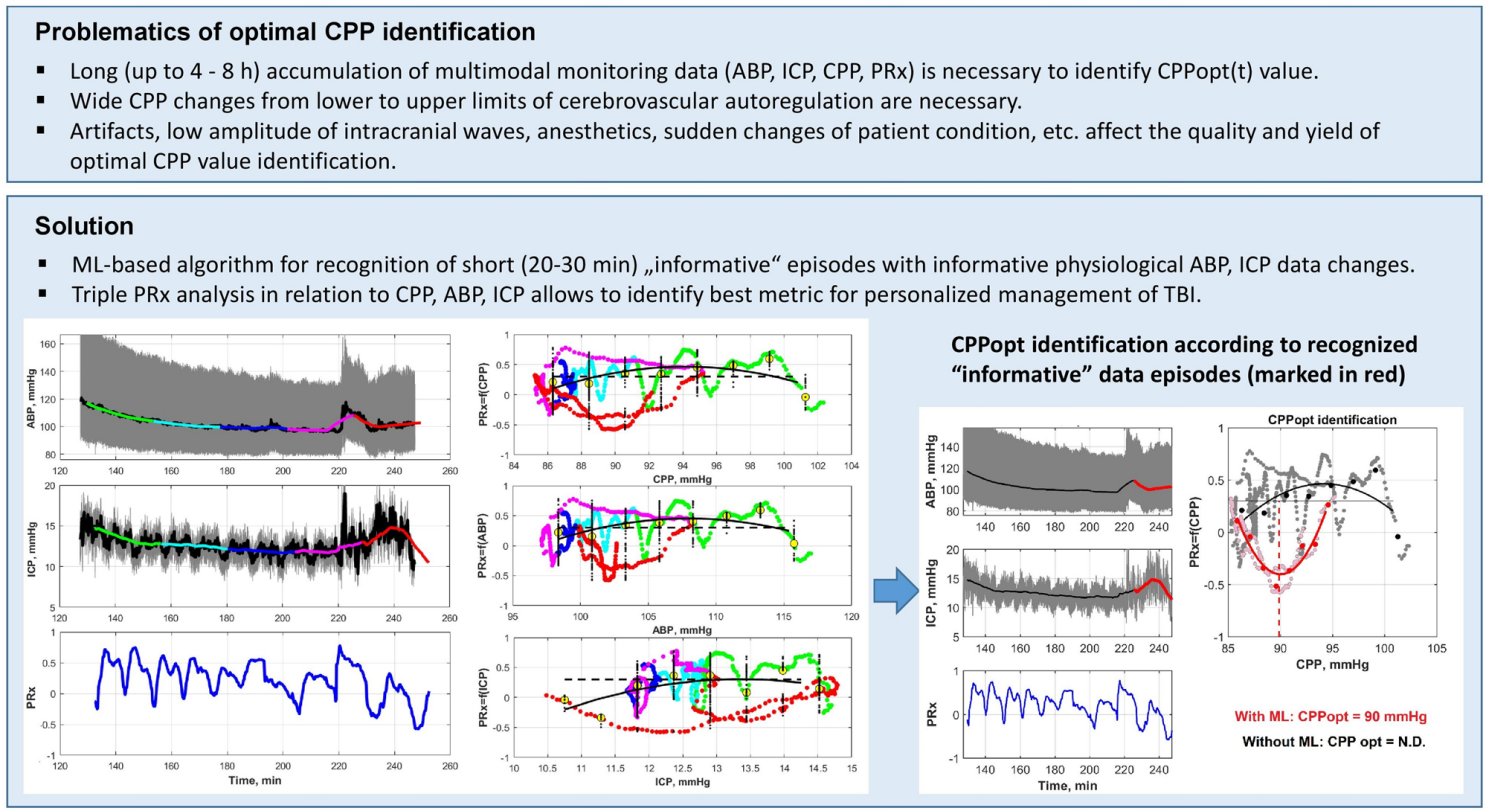
Problematics of CPPopt value identification and research idea. Machine learning (ML)-based algorithms are applied to recognize the informative physiological ABP/ICP variations within short (20–30 min) intervals and use these data episodes for the timely identification of optimal value (or lower/upper CA limits) in CPP, ABP or ICP data. CPPopt(t) value is identified according to recognized “informative” data episodes (marked in red) as a minimum point of “U-shape” approximation in PRx = f(CPP) graph.

In our research, the idea is to show the possibility of improving the existing CPPopt identification algorithms by applying machine learning (ML) methods, to shorten the time needed to identify optimal and critical values for CPP/ABP/ICP as well as to choose an appropriate management strategy for timely CA status stabilization. The problematics and main idea of this research are presented in Fig. 1.

Machine learning (ML) techniques, such as support vector machines, decision trees, and artificial neural networks, have already been applied for the development of models for predicting the outcome of TBI patients^21–24^, predicting the increase in ICP^25^, and for the rejection of monitoring artifacts in real-time^26^.

A literature analysis has shown that ML methods are still not used in CPPopt identification algorithms in order to reducethe CPPopt identification time as much as possible, resulting in more effective CPPopt-targeted treatment for TBI patients.

## Methods

### Patient data

The retrospective analysis of multimodal physiological monitoring data of 87 severe TBI patients with different outcomes (67.8% survival, 32.2% fatality), ages (18–86 years), and sex (67% male, 33% female) contributed to this research. The high-resolution continuous monitoring data of ICP(t), ABP(t), CPP(t), and PRx(t) were registered and analyzed using ICM + software. The patients’ monitoring time varied from 6 h to 10 days. The data were collected in the ICU of the Republican Vilnius University Hospital and Lithuanian University of Health Science Kaunas Clinics from 2013–2018.The inclusion criteria were as follows: TBI patients with invasive ICP and ABP sensors, age > 18 years, and a minimal time of CA monitoring data 6-h. Patients or their relatives signed a consent form. Detailed demographic characteristics and average values of the monitored clinical data of the TBI patients included in this analysis are listed in Table 1.

**Table 1:** Demographic and multimodal monitoring data of TBI patients.

	Outcome			p value	
	Unfavorable		Favorable	Favorable vs. unfavorable	Fatal vs. survival
	(GOS = 1)	(GOS = 2, 3)	(GOS = 4, 5)		
Number of patients, number, (%)	27 (31)	17 (19.5)	43 (49.5)	–	–
Sex (M/F)	20/7	13/4	34/3	–	–
Age, years	48.0 (15.0)	45.7 (15.1)	34.0 (14.5)	< 0.001	0.006
GCS	5 (3)	4 (2)	7 (3)	0.001	0.099
Average ICP, mmHg	26.9 (26.3)	14.0 (3.9)	13.8 (5.0)	0.097	0.061
Average ABP, mmHg	44.5 (51.5)	70.9 (9.7)	69.8 (12.6)	0.230	0.060
Average CPP, mmHg	71.4 (27.6)	84.4 (8.4)	83.6 (10.0)	0.268	0.099
Average CPPopt, mmHg	71.9 (26.8)	85.5 (9.8)	82.8 (8.6)	0.425	0.083
Average ΔCPPopt, mmHg	−8.24 (18.5)	−0.55 (5.96)	1.12 (5.97)	0.004	0.024
Average PRx	0.38 (0.32)	0.04 (0.23)	0.09 (0.13)	0.010	< 0.001

Here, ICP is the intracranial pressure, ABP is the arterial blood pressure, CPP is the cerebral perfusion pressure, PRx is the pressure reactivity index, ΔCPPopt is declination of CPP from CPPopt, GCS is the Glasgow Coma Scale, GOS is the Glasgow Outcome Score after 6 months. Favorable outcomes are good recovery (GOS = 5) and moderate disability (GOS = 4), and unfavorable outcomes are severe disability (GOS = 3), vegetative state (GOS = 2), or death (GOS = 1). The p values are calculated by using the Mann–Whitney U test for differences between the groups with favorable (GOS = 4,5) and unfavorable (GOS = 1,2,3) outcomes and between the groups with survival (GOS = 2,3,4,5) and fatal (GOS = 1) outcomes (significant if p < 0.05). Age and average ICP, ABP, CPP, PRx, ΔCPPopt values expressed by mean with standard deviation (in brackets). The GCS is expressed as the median and interquartile range in brackets.

### Ethics approval and consent to participate

The collection of clinical data used in this study was performed in accordance with study protocols approved by the Ethics Committee of Vilnius and Kaunas (Lithuania) Regional Biomedical Research Ethics Committees (Protocol Nos. 158200-06-498-145, 158200-16-854-364, 2016-07-12, 158200-15-801-323, 2015-10-06 and BE-2-6, 2015-01-06). All research and patient treatment were performed in accordance with the Lithuanian guidelines of head injury diagnostics and management (2010) and the national methodical recommendations for the diagnosis and treatment of brain injuries (2017). The participants and/or their legal guardians signed an informed written consent for the use of their anonymized clinical data in this retrospective analysis.

### Software for ICU multimodal monitoring data analysis

The software for retrospective ICU multimodal monitoring data analysis was developed using the MATLAB 9.9 (R2020b) toolbox. This software provides the possibility to review retrospectively monitored ABP(t), ICP(t), CPP(t) and PRx(t) data within a 0.5-h to 4-h selected time window and to analyze these data by plotting the three graphs: PRx = f(CPP), PRx = f(ABP), and PRx = f(ICP).

**Figure 2:**
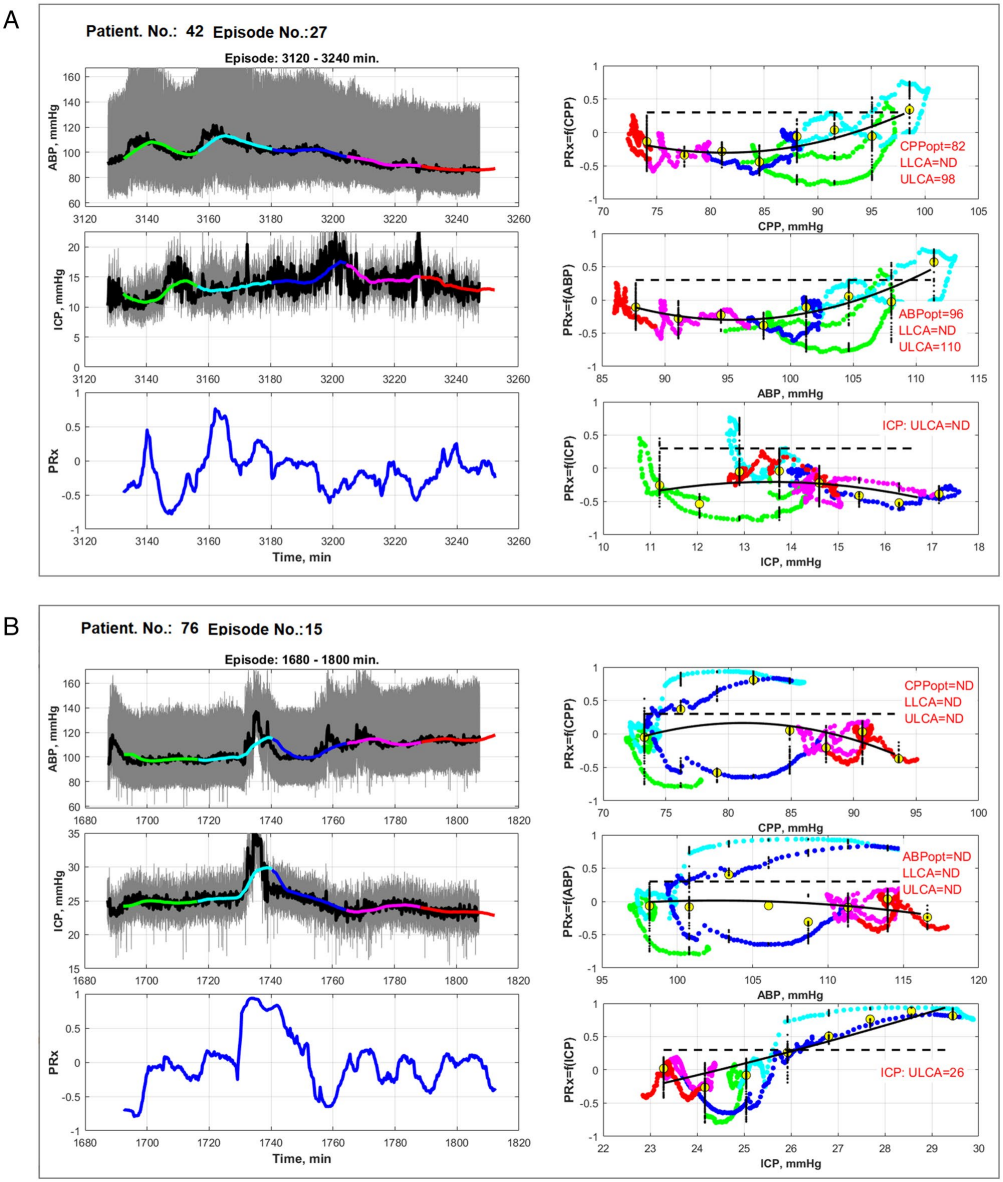
Software tool for ICU multimodal monitoring data analysis and CPPopt identification. The contribution of ABP and ICP color-marked episodes (i.e., green, cyan, blue, pink and red) to the formation of quadratic approximation curves can be analyzed by including or removing them from the PRx = f(CPP), PRx = f(ABP), and PRx = f(ICP) graphs. Color-marked segments are selected from software menu by assigning segments into “artifact-free”/”artifact-disturbed” or “informative”/”noninformative” classes which are required to train and test the ML algorithms. The example in (**A**) represents the case when CPPopt and ABPopt together with the upper limits of autoregulation (ULCA) are identified, thus demonstrating that CPPopt-targeted treatment can be applied for the personalized management to control intact CA. The example in (**B**) shows that CPPopt identification can be disturbed due to an ICP increase. However, the detected ULCA for ICP shows that ICP increase above 26 mmHg leads to impaired CA. Here ND is “not detected”.

Each selected monitoring episode was divided into five color-marked segments that could be manually included or excluded into into PRx = f(CPP), PRx = f(ABP), PRx = f(ICP) graphs, thus allowing for the separate analysis of the influence of each of the color-marked segments on CPPopt value identification. The colors (green, cyan, blue, pink, and red) were assigned to each segment to separate and distinguish them along with the time window (Fig. 2).

The software is used to fit the quadratic polynomial over PRx = f(CPP), PRx = f(ABP), and PRx = f(ICP) graphs to obtain “U-shape” approximations. The minimum points of the “U-shape” curves are calculated for the PRx = f(CPP) and PRx = f(ABP) graphs and presented as the CPPopt and ABPopt values. Additionally, the lower limit of cerebral autoregulation (LLCA) and upper limit of cerebral autoregulation (ULCA) values were calculated from the PRx = f(CPP) and PRx = f(ABP) graphs at the points where the approximation curve crosses PRx = 0.3 level, assuming that outside these limits, CA is impaired^15,27,28^. A threshold value of PRx =  + 0.3 was chosen according to previous studies, in which it has been shown that the average value of PRx that exceeds + 0.3 is associated with a fatal outcome for severe TBI patients. Therefore, this value can be used for the determination of LLCA and ULCA in “U-shape” approximation^15,27,28^. ULCA only is calculated for the PRx = f(ICP) graph considering, that the optimal ICP calculation is meaningless in TBI cases.

The proposed triple graphical decomposition of PRx dependencies on CPP, ABP, and ICP allows for an easier interpretation of the clinical phenomena and aids in the decision of which factor (ABP, ICP, or CPP) should be used to stabilize the CA status and the critical limits (LLCA and ULCA) of these parameters.

Examples of windows in the developed software are shown in Fig. 2A,B. Figure 2A represents the case when CPPopt and ABPopt are identified together with ULCA, thus demonstrating that ABP should be used as a driving parameter to keep the CA intact. Figure 2B shows an example when CPPopt identification is impossible due to the disturbed U-shape by an increase in ICP event. However, this event allows us to detected ULCA in ICP data, showing that ICP increase above 26 mmHg leads to impaired CA.

### Annotation of monitored episodes

The developed software includes an annotation function that enables the assignment of the monitored segments as “informative”/“noninformative” and “artifact-free”/“artifact-distorted” classes. The “informative” segments are assigned as the segments that reflect physiological reactions of vasogenic slow intracranial B-waves^16,29^ and contribute to the formation of the desired “U-shape”, and therefore, can be used for the identification of CPPopt (or ABPopt), LLCA, or ULCA. There is an additional option available to classify the segments that represent “artifact-free” or “artifact-distorted” classes. Segment annotation is performed by selecting the corresponding colored segments and, including or removing them from the calculations. All patients data annotation was performed by two clinical physicists.

In creating an ML-based algorithm, we used 2-h data window episodes that were divided into five segments of 24-min for episode deconstruction.

An additional software function is the ability to annotate five different clinical situations according to the possibility of applying different management strategies for intact CA status stabilization:

1. CPP-guided management (when the U-shape or LLCA/ULCA exist in PRx = f(CPP)).
2. ABP-guided management (when the U-shape or LLCA/ULCA exist in PRx = f(ABP)).
3. ICP-guided management (when ULCA exists in PRx = f(ICP)).
4. CA is intact (PRx < 0); therefore, no specific treatment is necessary.
5. CA is critical (when the mean PRx is above 0.3 for all CPP values); this requires immediate intervention.

### Machine learning (ML) model

The supervised ML model based on support vector machines (SVMs)^30^ was applied to create multimodal monitoring data analysis algorithms using R: A Language and Environment for Statistical Computing (e1071 package).

Two datasets containing 324 2-h episodes of 1620 “artifact-free”/“artifact-distorted” annotated segments and 255 2-h episodes of 1275 “informative”/”noninformative “ annotated segments were formed and used for training the ML models to detect “artifact-distorted” as well as “informative” episodes.

The input data “X1” for the artifact detection model was the set of ABP- and ICP-related parameters from the annotated segments and includes the difference between systolic and diastolic values, the derivative of this difference, and the duration of events when this difference is below the specifically selected values (5 and 10 mmHg). The output data “Y1” for the artifact detection model were the annotated 24 min length segments labeled as “1” for “artifact-free” segments and “0” for “artifact-distorted” segments.

The input data “X2” for “informative” segment recognition was the set of ABP- and ICP-related parameters, including segments of the standard deviations of ICP and ABP values filtered with 1-, 2-, 3-, and 4-min moving average time windows and the mean of values of ABP and ICP. The output data “Y2” for the informative segment recognition model were the annotated 24-min length segments labeled as “1” for “informative” segments and “0” for “noninformative” segments.

An SVM with a radial basis function (RBF) kernel was used to create ML models for the recognition of “informative “ and “artifact-free” segments and CPP-, ABP- or ICP-guided management strategies. Distinguishing feature of kernel function application in the SVM algorithm is equivalent to the possibility of recognizing nonlinearly separated classes (for example, “informative”/“noninformative” segments) by separating them in a multidimensional hyperspace. The soft-margin and mapping to the feature space by the RBF kernel are applied to separate nonlinearly distributed classes^30^.

Parameters C (regularization cost) and γ (gamma, the width of an RBF) were selected using a grid search. Since the range of these parameters is unknown, in the initial research, the default values (C = 1 and γ = reciprocal of input variable dimension) were adjusted by exponentially growing factors (i.e., 1/32, 1/16, 1/8, ¼, ½, 1, 2, 4, 8, 16 and 32). The grid search trained the SVM model with each pair (C, γ) in the Cartesian product of these two sets by evaluating model performance using tenfold cross-validation to find the optimal combination of C and γ according to the highest accuracy of ML models. Since the cross-validation technique uses the separate parts of the sample for model building and for accuracy estimation, the selection of overfitted models is avoided^31^.

**Figure 3:**
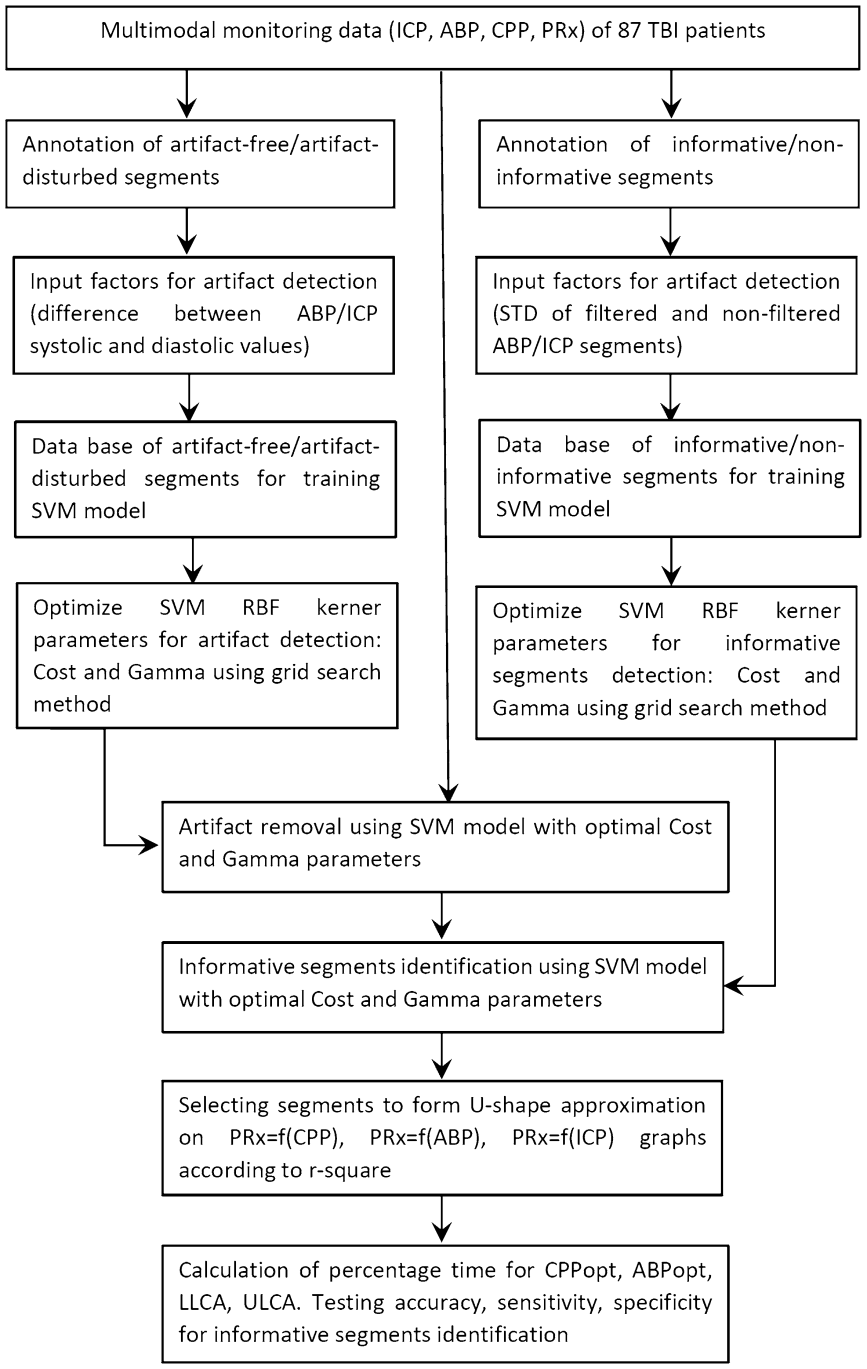
Workflow diagram of multimodal monitoring data analysis using ML models for CPPopt, ABPopt, LLCA, and ULCA identification.

All patients’ monitoring data were processed to remove the artifact-distorted episodes and to identify “informative” segments that could be used for CPPopt (or ABP opt) and LLCA and ULCA identification. Additional “artifact-free” segments were analyzed according to their contribution to the quality of the U-shape approximation curve and were included in the calculation of CPPopt (or ABP opt) and LLCA and ULCA if they increased the R^2^ of the approximation fitting curve. The workflow diagram of multimodal monitoring data analysis using ML models is shown in Fig. 3.

### Model testing

The patients’ multimodal monitoring data used for creating and testing developed models contained 2410 2- episodes consisting of 12,050 annotated segments which included 655 “artifact-distorted” and 11,395 “artifact-free” segments (imbalance ratio 0.945), and 8969 “informative” and 3081 “noninformative” (imbalance ratio 0.74432) episodes.

The developed models were tested by determining the accuracy, sensitivity and specificity to recognize “artifact-distorted” and “informative” data monitoring episodes as well as to diagnose which physiological parameter (CPP, ABP or ICP) could be used for keeping CA intact by comparing the performance of ML-based models to a standard model (i.e., a model without ML).

The overall model performance was tested by calculating the percentage of time (also called as yield^18,19^) when the values of CPPopt, ABPopt or LLCA/ULCA were identified in 2-h monitoring window.

## Results

**Figure 4:**
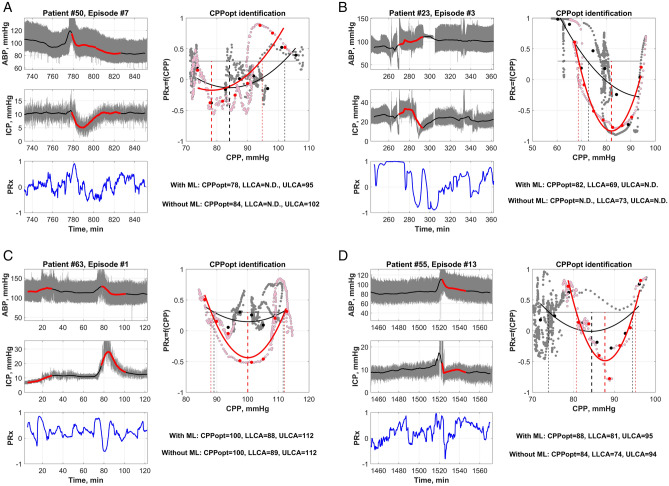
Examples of CPPopt identification according to informative ABP and ICP episodes (marked in red). The PRx = f(CPP) graph demonstrated the contribution to informative data points (pink) and noninformative data points (gray) to the formation of the U-shape approximation and CPPopt identification results. The red curve in the PRx = f(CPP) graph represents the U-shape approximation when ML is applied, and the black curve is the approximation when ML is not applied. These results show that short informative episodes (20–30 min) with informative physiological ABP and ICP data changes are sufficient to obtain the U-shape and identify the CPPopt value. (**a**)—Without ML (black color) CPPopt = 84 mmHg, LLCA is not detected and ULCA = 102 mmHg. With the ML algorithm (red color) CPPopt = 78 mmHg, LLCA not detected and ULCA = 95 mmHg. (**b**)—Without ML (black color) CPPopt is not detected, LLCA = 73 mmHg and ULCA is not detected. With the ML algorithm (red color) CPPopt = 82 mmHg, LLCA = 69 mmHg and ULCA is not detected. (**c**) Without ML (black color) CPPopt = 100 mmHg, LLCA = 89 mmHg and ULCA = 112 mmHg. With the ML algorithm (red color) CPPopt = 100 mmHg, LLCA = 88 mmHg and ULCA = 112 mmHg. (**d**) Without ML (black color) CPPopt = 84 mmHg, LLCA = 74 mmHg and ULCA = 94 mmHg. With the ML algorithm (red color) CPPopt = 88 mmHg, LLCA = 81 mmHg and ULCA = 95 mmHg. Here ND is “not detected”.

The determined SVM model’s diagnostic characteristics (accuracy, sensitivity, specificity and area under the curve) for automatically recognizing “artifact-distorted” segments, “informative” segments and the identification of guiding factors (CPP, ABP or ICP) for personalized management are listed in Table 2. Examples of the identification of patient-specific CPPopt values by using the informative segments detected with the developed ML-based algorithms are shown in Fig. 4.

**Table 2:** Model accuracy, sensitivity, specificity to predict “artifact-disturbed” segments, and “informative” episodes and to recognize cases of CPP-, ABP- or ICP-guided management for personalized TBI treatment.

Detected parameter	Accuracy, CI	Sensitivity	Specificity	AUC	Cohen’skappa	Imbalance ratio
**Artifact detection model**						
Artifact-disturbed segment (within training datasets)	0.892 (0.876–0.907)	0.963	0.657	0.891	0.673	0.766
Artifact-disturbed segment (overall data)	0.965 (0.962–0.969)	0.971	0.849	0.976	0.707	0.945
**Informative episodes detection model**						
Informative episode (within training datasets)	0.719 (0.694–0.744)	0.737	0.7035	0.773	0.439	0.532
Informative episode (overall data)	0.790 (0.784–0.799)	0.940	0.352	0.646*	0.346	0.744
**Model for CPP-guided management recognition**						
CPP-guided management	0.6705 (0.651–0.690)	0.681	0.662	0.739	0.341	0.527
**Model for ABP-guided management recognition**						
ABP-guided management	0.686 CI: (0.667–0.705)	0.514	0.809	0.742	0.333	0.586
**Model for ICP-guided management recognition**						
ICP-guided management	0.774 (0.757–0.790)	0.305	0.892	0.7490	0.300	0.735
**Model for critical patients’ condition recognition**						
Critical condition	0.946 (0.936–0.955)	0.865	0.972	0.982	0.850	0.761
**Model for intact CA status recognition**						
Intact CA condition	0.902 (0.890–0.914)	0.771	0.940	0.952	0.715	0.779

Here, CA is the cerebrovascular autoregulation, ICP is the intracranial pressure, ABP is the arterial blood pressure, CPP is the cerebral perfusion pressure, AUC is area under the curve, , and CI is confidence interval at the 95% level. Imbalance is the ratio of data samples between classes in the datasets.

* AUC is calculated assuming output classes with a fixed probability.

**Figure 5:**
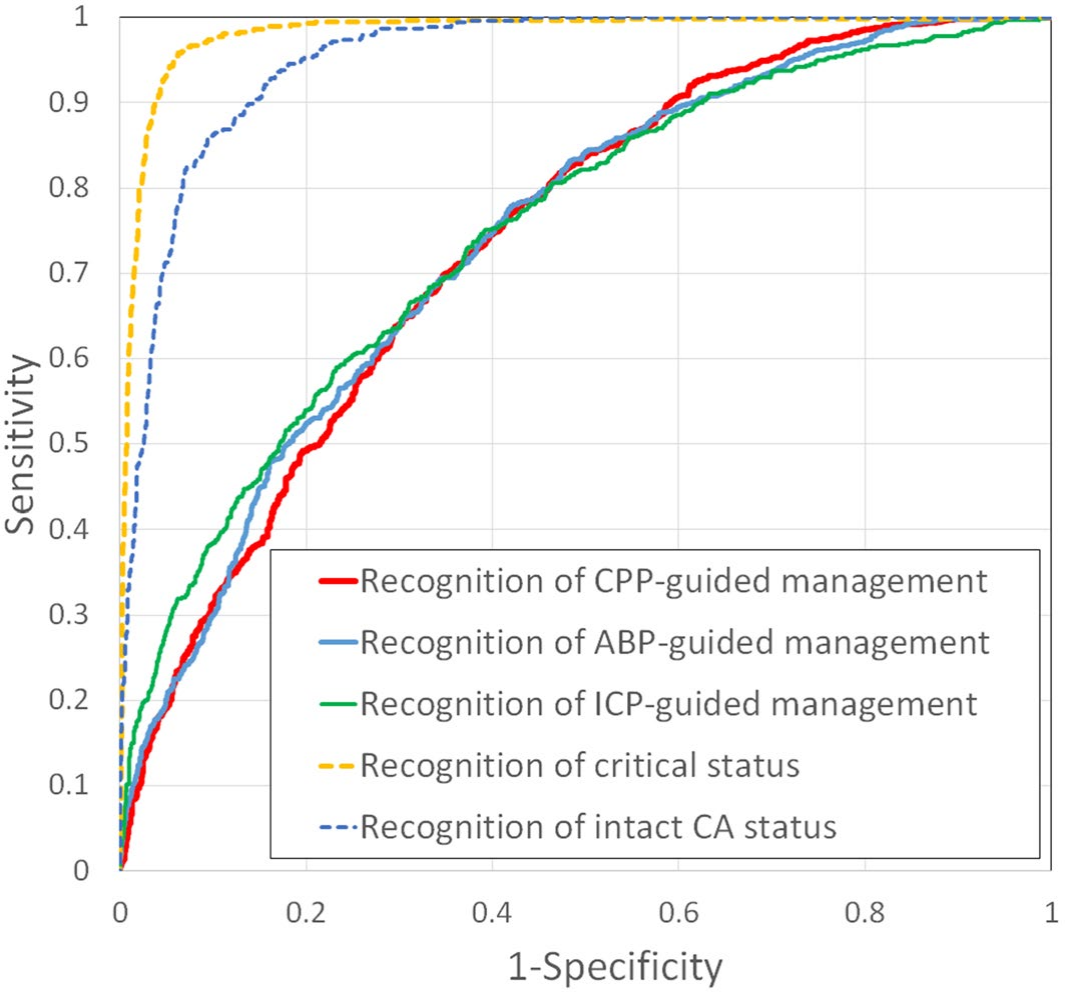
Representation of the diagnostic ability of the developed ML model to recognize CPP-, ABP- or ICP-guided management, as demonstrated by the receiver operating characteristic (ROC) curves. ROC characteristics for recognition CPP-guided management: sensitivity 68%, specificity 66%, AUC 0.74; ABP-guided management: sensitivity 51%, specificity 81%, AUC 0.74, ICP-guided management: sensitivity 31%, specificity 89%, AUC 0.75; recognition of critical status: sensitivity 87%, specificity 97%, AUC 0.98; recognition of intact CA status: sensitivity 77%, specificity 94%, AUC 0.95.

The overall model performance was tested by calculating the percentage time when the identification of CPPopt, ABPopt, and LLCA, ULCA including (ULCA for ICP) was possible. The results of the percentage time calculation in the cases with and without using ML models are listed in Table 3. The receiver operating characteristic (ROC) analysis showing the predictive capabilities of ML models to recognize appropriate CPP, ABP or ICP-guided management and to identify cases of critical or intact CA statuses are presented in Fig. 5.

**Table 3:** Percentage time of multimodal monitoring cases when CPPopt, ABPopt, and LLCA/ULCA are identified.

Multimodal monitoring cases	Percentage time when identified, % (without ML)	Percentage time when identified, % (with ML)
CPPopt identified	32.07	34.69
ABPopt identified	25.56	26.43
ICP(ULCA) identified	21.24	21.29
CPP (Opt or LLCA/ULCA identified	55.77	58.38
ABP (Opt or LLCA/ULCA) identified	50.87	51.99
ABP (Opt or LLCA/ULCA) or ICP (ULCA) identified	68.37	69.46
CPP or ABP (Opt or LLCA/ULCA) identified	74.94	76.89
ABP or CPP (Opt or LLCA/ULCA) or ICP (ULCA) identified	77.59	79.21
Intact CA status	12.16	12.28
Critical CA status	3.53	4.19

Here, CA is the cerebrovascular autoregulation, ICP is the intracranial pressure, ABP is the arterial blood pressure, CPP is the cerebral perfusion pressure, ULCA is the upper limit of cerebrovascular autoregulation, LLCA is the lower limit of cerebrovascular autoregulation, Opt is the optimal value, and ML represents machine learning.

## Discussion

The aim of this research is to show the potential to improve the performance of CPPopt-based personalized management by identifying the optimal and critical values of CPP, ABP, or ICP-guided treatment and to choose the appropriate treatment strategy for normalizing the intact CA in a timely manner.

Many clinical studies have demonstrated that time spent below CPPopt is harmful, especially when CA is impaired^1,5,8,15^. A decrease in CPP below LLCA causes hypoperfusion and is fatal for TBI patients, while an increase in CPP above ULCA causes hyperperfusion and is associated with severe disability^8^. However, personalized patient treatment is possible only when CPPopt is identified in an appropriate time, allowing the implementation of CA-guided management. Failure to identify the CPPopt value is associated with the occurrence of delayed cerebral ischemia in aneurysmal SAH patients^32^.

The occurrence of different factors during the long data accumulation time window, such as artifacts or nonphysiological data variations, sudden changes in the patient’s clinical condition, the influence of anesthetics or various pharmaceutical therapies, can distort the monitoring data, making CPPopt value identification impossible^33,34^. Additionally, CPPopt cannot be calculated when the CA status is completely impaired (when PRx value is continuously above critical threshold 0.3), when CA is intact (PRx < 0), or when the CPP varies within a small range that does not reach the LLCA or ULCA. In some cases, the identification of CPPopt and the interpretation of CA monitoring results might differ between clinicians^33^. CPPopt curves may be disturbed by many factors, meaning that some degree of physician assessment and interpretation of the autoregulated data is necessary^34^.

In this study, we proposed approaches ways to improve the performance of the CPPopt identification method.

First, to achieve the necessary time accuracy for CPP opt value identification, we used a 2-h time window divided into 5 segments, each with a 24-min length. We applied ML methods and trained them to recognize data segments that contain informative physiological slow variations in ABP and ICP signals and to use them as key segments to identify the CPPopt value or CA limit. Additional “artifact-free” segments were also added in the calculation if they increased the r-square of the U-shape approximation curve.

Second, to minimize the time required to choose an appropriate decision regarding patients’ treatment, it is not necessary to know the exact CPPopt value. In many cases, it is sufficient to know the CPP limits (ULCA or LLCA) at which CA starts to deteriorate. For this reason, it is not necessary to wait a few hours until the CPP changes in a wide enough range to obtain “U-shape” approximation curve. It is sufficient to identify “informative” episodes with CPP (or ABP) changes to determine the direction of CPP (or ABP/ICP) changes for driving CA into intact status.

Third, we suggested representing PRx monitoring data separately in relation to ABP, ICP, and CPP by plotting three graphs PRx = f(CPP), PRx = f(ABP), and PRx = f(ICP). Such representations could help physicians choose the appropriate individualized management by determining which factor, ABP, CPP, or ICP, is a key parameter for normalizing CA status, specifically in complex scenarios when CPPopt is not identified. For example, simultaneous dynamics in ICP and ABP can lead to cases where the identification of CPPopt is not possible due to ICP influence. ICP management should be applied to normalize CA status if ULCA is identified for ICP (Fig. 2b).

Moreover, we found these triple graph representations can increase the yield when choosing the appropriate ABP, ICP, or CPP parameters to normalize the CA status.

Our study showed that the obtained yield for CPPopt identification using a 2-h monitoring window is approximately 32–34%. However, this yield can be increased up to 66–68% when including the cases of detected ULCA or LLCA in the CPP data (Table 3). The yield for ABPopt identification is 25–26%, however, this yield increased up to 55–58% when the cases of ULCA or LLCA identification in ABP data were included. Moreover, by combining all cases when CPPopt, ABPopt, or LLCA/ULCA are identified and including the cases when ICP(ULCA) is identified, an overall yield for choosing the appropriate guided TBI management up to 75% (without ML) and 79% (with ML) was achieved. It was also shown that in 12% of cases, when the CA was continuously normal (PRx < 0), and in 3% of cases when CA was continuously completely disturbed (PRx > 0.3), the identification of CPPopt, ABPopt, or LLCA/ULCA values was impossible.

In this study, we focused on the possibilities of improving the performance of the existing CPPopt targeted management strategies by applying ML methods, which aim to recognize informative data segments and clinical situations when CPPopt identification is possible. The initial test of ML models on the reference datasets of the “informative”/”noninformative” and “artifact-free”/”artifact-disturbed” segments showed a slightly higher accuracy in recognizing the appropriate informative data episodes needed for CPPopt, ABP opt or ULCA/LLCA identification from limited time data by applying the SVM method with an RBF kernel compared to other methods (SVMs without kernel, decision trees, boosting (using the XGBoost library), random forests, and artificial neural networks). Analysis of the applied models showed that the main factors affecting ML-model performance were the initial imbalance of class sizes and subjective data annotation when assigning data to classes.

The developed ML-based algorithms provide a possibility to achieve an accuracy of up to 96% for detecting artifacts, 79% for recognizing informative episodes, and up to 67–77% for detecting appropriate management for TBI treatment. Although these numbers appear high, real improvements in the accuracy to detect or recognize specific clinical situations (compared to the initial imbalance in the datasets) were + 2.7% for detecting artifacts, + 4.5% for recognizing informative data segments, + 14.4% for detecting CPP-guided management cases, + 10% for detecting ABP-guided management cases and + 3.9% for detecting ICP-guided management cases, + 10.4% for recognizing intact CA cases (when no specific therapy is necessary), and + 12.3% for recognizing critical CA cases. A relatively small improvement in classification accuracy is associated with the relatively high initial imbalance of data classes within datasets (~ 0.7–0.8), which impacts the performance of ML-based method. Therefore, real accuracy characteristics for developed models can be estimated only by performing further prospective studies.

An additional limitation is that the dataset preparation and data annotation require specific knowledge and experience in the treatment of TBI patient and the perception of CPPopt-guided personal TBI management. The annotations of data monitoring segments, i.e., assigning segments to specific classes as well as grouping clinical events according to the most suitable ABP, ICP or CPP management, were performed by two experienced clinical physicists from two different medical centers. These physicists performed data analysis on only of the patients treated in the ICU departments of their respective institutions. Although both annotators are experienced in brain physics and TBI treatment, the accuracy of the developed ML models is directly related to the annotators’ personal experience and their subjective evaluation of each specific case.

Despite these limitations, it was demonstrated that the developed ML-based algorithms allow us to recognize informative physiological ABP/ICP variations within 24 min intervals and use them for a timely identification of optimal value or CA limits determination in CPP, ABP or ICP data. The examples provided in Fig. 4 demonstrate that the developed ML-based algorithm could be used to identify CPPopt (or LLCA/ULCA) values even from a single 24-min data segments (which is 5-times faster compared to the existing methods that require a minimal 2-h monitoring time for data accumulation) in the cases where informative physiological ABP/ICP variations or slow waves were detected. An additional impact of the developed ML-based algorithms is the ability to identify the appropriate individualized CPP-, ABP- or ICP-guided management from a 2-h time window. A percentage time of up to 79% (which is close to the ~ 90% yield obtained by using a multiwindow weighting algorithm approach based on CPPopt identification using 2- to 8-h windows^18,19,33^) was achieved when CPPopt, ABPopt, or LLCA/ULCA were identified during the full monitoring time.

Currently, there are no ML-based approaches that are directly used in the personalized treatment of TBI patients or in outcome prediction. The modern ML-based approaches that have been implemented for TBI management, such as semisupervised learning methods (i.e., kernel spectral regression and SVM), for the detection of ICP signal alarms^35^ and for the early warning of intracranial hypertension^25,36^ are more theoretical-based models, the implementation of which requires clinical validations in precision medicine. Recent studies have shown that the methods for TBI patient outcome prediction outperform traditional regression approaches and should be rigorously validated to ensure their applicability to new populations^24^. ML-based approaches are more feasible for removing artifact from ICU monitoring data, which are especially apparent in ABP data^26,37,38^. Such applications of ML methods allows us to improve the quality of multimodal monitoring data that is needed for making treatment decisions. Our idea was to supplement this concept by developing algorithms for both artifact detection and informative data segments recognition. An additional finding was obtained by separately analyzing the impact of CPP, ABP and ICP on CA status. This type of analysis also allows us to determine which cerebral metrics (CPP, ABP or ICP) could be used as a key factor for maintaining intact CA.

Therefore, the algorithms developed in this study can be used as machine-based advisors in complex clinical scenarios, helping clinicians recognize both informative segments that could be used for CPPopt (or ABPopt) identification as well as for the appropriate selection of cerebral metrics for CA status normalization.

## Conclusions

A retrospective study of 87 severe TBI patients with multimodal monitoring data showed the potential to improve algorithms for personalized CPPopt values by reducing the monitoring data accumulation time.

The developed ML-based algorithms allow us to recognize the informative physiological ABP/ICP variations within 24 min intervals and use these segments for the timely determination of optimal value or CA limits identification in CPP, ABP or ICP data. It has also been shown that ML-based identification of appropriate individualized CPP-, ABP- or ICP-guided management from a 2-h monitoring window is possible in 79% of the full monitoring time of the studied population data.

Prospective clinical studies are guaranteed to prove the efficiency of the developed algorithms.

## Data Availability

The clinical data (anonymized) used in this study are available from the corresponding author, V.Pe. upon request: https://doi.org/10.5281/zenodo.5079804.
